# Investigating the utility of human embryonic stem cell-derived neurons to model ageing and neurodegenerative disease using whole-genome gene expression and splicing analysis

**DOI:** 10.1111/j.1471-4159.2012.07825.x

**Published:** 2012-08

**Authors:** Rickie Patani, Patrick A Lewis, Daniah Trabzuni, Clare A Puddifoot, David J A Wyllie, Robert Walker, Colin Smith, Giles E Hardingham, Michael Weale, John Hardy, Siddharthan Chandran, Mina Ryten

**Affiliations:** * Anne McLaren Laboratory for Regenerative Medicine, University of CambridgeCambridge, UK; † Department of Clinical Neurosciences, Cambridge Centre for Brain Repair, University of CambridgeCambridge, UK; ‡ Department of Molecular Neuroscience, UCL Institute of NeurologyLondon, UK; § Department of Genetics, King Faisal Specialist Hospital and Research CentreRiyadh, Saudi Arabia; ¶ Centre for Integrative Physiology, University of EdinburghEdinburgh, UK; ** MRC Sudden Death Brain Bank Project, Department of Neuropathology, University of EdinburghEdinburgh, UK; †† Department of Medical & Molecular Genetics, King’s College London, Guy’s HospitalLondon, UK; ‡‡ Euan MacDonald Centre, MRC Centre for Regenerative Medicine, Centre for Clinical Brain Sciences, University of EdinburghEdinburgh, UK

**Keywords:** dopaminergic neurons, hESC, human brain, microarray, neurodegenerative disease, splicing

## Abstract

A major goal in regenerative medicine is the predictable manipulation of human embryonic stem cells (hESCs) to defined cell fates that faithfully represent their somatic counterparts. Directed differentiation of hESCs into neuronal populations has galvanized much interest into their potential application in modelling neurodegenerative disease. However, neurodegenerative diseases are age-related, and therefore establishing the maturational comparability of hESC-derived neural derivatives is critical to generating accurate *in vitro* model systems. We address this issue by comparing genome-wide, exon-specific expression analyses of pluripotent hESCs, multipotent neural precursor cells and a terminally differentiated enriched neuronal population to expression data from post-mortem foetal and adult human brain samples. We show that hESC-derived neuronal cultures (using a midbrain differentiation protocol as a prototypic example of lineage restriction), while successful in generating physiologically functional neurons, are closer to foetal than adult human brain in terms of molecular maturation. These findings suggest that developmental stage has a more dominant influence on the cellular transcriptome than regional identity. In addition, we demonstrate that developmentally regulated gene splicing is common, and potentially a more sensitive measure of maturational state than gene expression profiling alone. In summary, this study highlights the value of genomic indices in refining and validating optimal cell populations appropriate for modelling ageing and neurodegeneration.

The ability to generate defined neuronal lineages from human embryonic stem cells (hESCs) offers an unprecedented opportunity to establish *in vitro* model systems of human neurodegenerative diseases. Embryonic stem cells predictably respond to developmental morphogenetic signals, thus permitting the generation of specific neuronal types to enable study of region-specific neurodegenerative conditions *in vitro* (Lee *et al.* 2000; Kim *et al.* 2002; Wichterle *et al.* 2002; Lang *et al.* 2004; Perrier *et al.* 2004; Schulz *et al.* 2004; Bouhon *et al.* 2006). However, most neurodegenerative diseases are age-dependent (Lees *et al.* 2009). Therefore, it is important to determine to what extent hESC-derived neurons, which represent a developmental model system, resemble their adult counterparts not only morphologically and physiologically but also in terms of gene expression.

Although neuronal cultures have been studied with regard to specific genes such as *MAPT* (Iovino *et al.* 2010), to our knowledge no attempt has been made to compare and map hESC-derived neurons to region-specific comparators from the foetal or adult brain in a genome-wide manner. In part this is because of the relative scarcity of genome-wide data from control post-mortem brain. Genome-wide gene expression analysis offers a powerful method to comprehensively characterize temporally distinct stages of lineage restriction. Recent advances in both microarray and RNA sequencing technologies have allowed examination of splicing patterns in parallel with overall expression levels and have yielded an additional level of complexity to emerging studies (Yeo *et al.* 2007; Salomonis *et al.* 2010; Wu *et al.* 2010; Fathi *et al.* 2011). Using Affymetrix Exon arrays and next generation RNA sequencing, alterations in expression levels and splicing patterns during neuronal differentiation from hESCs have been demonstrated (Yeo *et al.* 2007; Wu *et al.* 2010; Fathi *et al.* 2011). However, this type of genome-wide transcriptome analysis has not previously been used to determine the maturational state of hESC-derived neuronal cultures compared to foetal and adult human brain samples. Generating electrophysiologically competent regionally defined neurons from hESCs does not, in itself, provide sufficient information regarding their maturational equivalence to their adult somatic counterparts. However, comprehensive genome-wide approaches may permit further meaningful analyses in this context. After all, it is now recognized that 90 per cent of genes expressed in the human brain are differentially regulated at the whole transcript or exon level across brain regions and/or time, making it possible to define regional and maturational cell states more precisely (Kang *et al.* 2011).

Against this background, we used Affymetrix exon arrays to investigate whole- genome gene expression and splicing to study the genomic equivalence of hESC-derived neural derivatives to foetal and adult human brain samples. Both gene and exon-level expression data generated from these arrays have been validated using TaqMan and Quantigene assays by ourselves and others (Yeo *et al.* 2007; Johnson *et al.* 2009; Kang *et al.* 2011; Trabzuni *et al.* 2011). Expression profiles were analysed using a variety of techniques including hierarchical clustering, principal component analysis (PCA), Gene Ontology analysis and gene set enrichment analysis (GSEA). By using a widely adopted midbrain dopaminergic differentiation protocol as representative of a clinically relevant population, we were also able to make inferences about how closely regionally defined (but heterogeneous) hESC-derived neuronal populations resembled their *in vivo* adult counterparts, given the growing use of such culture systems to model neurodegenerative disease. The major finding of our analysis was that hESC- derived neurons more closely resemble foetal brain and further that developmental stage is genomically a more significant influence than regional identity. In addition, we demonstrate that developmentally regulated splicing is a common finding and that splicing indices may be a more accurate measure of maturational state than gene level expression alone. These findings have significant implications for studies aiming to recapitulate an adult neurodegenerative disorder using human pluripotent stem cells, and provide a robust platform for studies aiming to uncover the molecular pathobiology of ageing and neurodegeneration.

## Materials and methods

### Human embryonic stem cell **(**hESC**)** culture and neural induction

The hESC lines H9 (WiCell Research Institute (Madison, WI, USA) and HuES9 (hES facility, Harvard University, Cambridge, MA, USA) were propagated in chemically defined medium (CDM) supplemented with FGF2, Activin (Harrington *et al.* 2006) and insulin (all at a concentration of 10 ng/mL) as an adherent culture system on a layer of mitotically inactivated mouse embryonic fibroblasts as previously described (Joannides *et al.* 2007; Patani *et al.* 2009). Human ESCs were enzymatically passaged in a 1 : 4 to 1 : 10 split ratio at the point of subconfluence. To generate neural precursor cells, hESCs were enzymatically dissociated, mechanically triturated then centrifuged before being washed in fresh medium. Cell aggregates were next suspended in chemically defined medium in 10-cm culture dishes on an orbital shaker. CDM constituents are as follows: 50% IMDM (Gibco, Rockville, MD, USA) plus 50% F12 plus Glutamax (Gibco), supplemented with 1.75 mM human recombinant insulin (Roche Molecular Biochemicals, Indianapolis, IN, USA), 0.38 mM transferrin (Roche), 450 μM of monothioglycerol (Sigma, St Louis, MO, USA), 10 μL/mL lipids (Sigma) and 5 mg/mL bovine serum albumin fraction V (Sigma). Mitogen (FGF2 at 10 ng/mL) was introduced to the cultures from day 8 for 8 days. At day 16, FGF2 was withdrawn and FGF8 (200 ng/mL) and a sonic hedgehog agonist puromorphamine (Li *et al.* 2008) at a concentration of 1 μM were simultaneously administered for midbrain dopaminergic neuronal specification. For terminal differentiation, midbrain precursors were plated onto poly-D-lysine/laminin coated coverslips and cultured in DMEM/2% B27/1%PSF, 10 ng/mL BDNF (R&D Systems) and 10 ng/mL GDNF (R & D Systems, Minneapolis, MN, USA) in the absence of mitogens.

### Reverse transcription-polymerase chain reaction **(**semi quantitative**)**

Total RNA was extracted from dissociated and washed cells using the RNeasy Mini Kit (Qiagen, Valencia, CA, USA) following the manufacturer’s instructions. In the case of terminally differentiated neuronal cultures, RNA extractions were performed at 59 days *in vitro*. cDNA was synthesized from 2 μg of RNA using Moloney murine leukaemia virus reverse transcriptase (Invitrogen, Carlsbad, CA, USA) and oligo-dT primers. Polymerase chain reaction (PCR) was carried out using Taq polymerase (Invitrogen). PCR products were separated on a 2% agarose gel and visualized with SYBR-Green (Invitrogen). Primer information is provided below:

PAX5 F: CCGAGCAGACCACAGAGTATTCA R: CAGTGACGGTCATAGGCAGTGG
ENGRAILED1 F: CTGGGTGTACTGCACACGTTAT R: TACTCGCTCTCGTCTTTGTCCT
ENGRAILED2 F: CCAGGTCTCGAAAACCAAAG R: CTACTCGCTGTCCGACTTGC

### Immunocytochemistry

Cells plated down on poly-D-lysine/laminin coated glass coverslips were fixed with 4% fresh paraformaldehyde for 20 min at 21°C and washed three times with PBS. Samples were next blocked for 1 h with 0.3% Triton/PBS/5% goat serum and then incubated overnight with primary antibody in 0.2% Triton/PBS/2% goat serum at 4°C. After three washes in PBS, secondary antibody (goat anti-mouse, Alexa Fluor 488 or 555, 1 : 1000) in PBS/Hoechst (1 : 4000) was next applied for 1 h. Primary antibodies included: NESTIN (1 : 500; Chemicon, Temecula, CA, USA), β-III-TUBULIN (1 : 500; Sigma-Aldrich), SYNAPSIN (1 : 500; Calbiochem, San Diego, CA, USA), ENGRAILED1 (1 : 50; Developmental Studies Hybridoma Bank, Iowa City, IA, USA), Musashi1 (1 : 500; Chemicon).

### Electrophysiological recordings

Whole-cell current-clamp and voltage-clamp recordings were made from midbrain dopaminergic neurons at (21 ± 2°C) using an Axopatch-1C amplifier (Molecular Devices, Union City, CA, USA) using methods as described previously (Baxter and Wyllie 2006; Soriano *et al.* 2008). Briefly, coverslips were transferred to a recording chamber perfused with an external recording solution composed of (in mM): 152 NaCl, 2.8 KCl, 10 HEPES, 2 CaCl_2_, 10 glucose pH 7.3 (320–330 mOsm). Patch pipettes were filled with a K-gluconate-based internal solution containing (in mM): 155 K-gluconate, 2 MgCl_2_, 10 Na-HEPES, 10 Na-PiCreatine, 2 Mg_2_-ATP and 0.3 Na_3_-GTP, pH 7.3 (300 mOsm). For current-clamp recordings, the external recording solution was supplemented with antagonists of ionotropic glutamate, GABA and glycine receptors (CNQX 5 μM; D-AP5, 50 μM, picrotoxin, 50 μM; strychnine 20 μM). For the recording of whole-cell AMPA- and NMDA-evoked currents and synaptically mediated glutamate receptor responses, the external solution was supplemented with picrotoxin (50 μM) and strychnine (20 μM). In all experiments where NMDA receptor-mediated responses were studied, a saturating concentration of the co-agonist, glycine (50 μM), was also added to the external recording solution. Miniature excitatory post-synaptic currents (mEPSCs) recorded in solutions supplemented with 300 nM tetrodotoxin (TTX), picrotoxin (50 μM), strychnine (20 μM) and MgCl_2_ (1 mM). Events were recorded for 5–10 min at a holding potential of −70 mV.

### Quantification and statistical analysis of cell cultures

A minimum of three biological repeats were utilized for all experiments unless otherwise stated in the text. Cell counts were performed across a minimum of five fields of view (approx. 90–350 cells per field) from a minimum of three biological experimental repeats across two different hESC lines. A *p* value of < 0.05 was considered statistically significant. Values are expressed as the mean ±SEM. The Mann-Whitney rank-sum test was used for non-parametric analysis using Graph-Pad Prism 4 (Graph-Pad Software Inc., San Diego, CA, USA).

### Human foetal and adult brain and samples

Data for foetal brain gene expression originated from Johnson *et al.*, Gene Expression Omnibus accession number GSE13344 (Johnson *et al.* 2009). Post-mortem adult substantia nigra samples were obtained from the MRC Sudden Death Brain and Tissue Bank at the University of Edinburgh (http://www.edinburghbrainbanks.ed.ac.uk). A detailed description of these tissue samples, isolation of mRNA, quality control and validation can be found in Trabzuni *et al.* (2011). A summary of all the samples used in this study is given in Supporting Information Table S1.

### RNA extraction and Exon Array processing

Total RNA was extracted from hESCs (*n* = 5), neural precursors (*n* = 4) and terminally differentiated neurons (*n* = 3) using the RNeasy kit (Qiagen, Crawley, UK) according to the manufacturer’s instructions. In the case of post-mortem adult brain samples (*n* = 57), total RNA was extracted using the miRNeasy kit (Qiagen) according to the manufacturer’s instructions. Following the evaluation of RNA quality using capillary electrophoresis (Agilent 2100 Bioanalyzer and RNA 6000 Nano Kit, Agilent Technologies, Wokingham, UK), 200 ng of total RNA was used as starting material for the cDNA preparation. All steps starting from the first and second strand cDNA synthesis, the *in vitro* transcription reaction to generate cRNA and the second round of cDNA synthesis were performed using the Ambion® WT Expression Kit (Ambion, UK) according to the manufacturer’s instructions. Samples were subsequently processed using the Affymetrix GeneChip Whole Transcript Sense Target Labelling Assay and hybridized to the Affymetrix Exon 1.0 ST Arrays following the recommended Affymetrix protocols. Hybridized arrays were scanned on GeneChip Scanner 3000 and visually inspected for hybridization artefacts. Affymetrix expression console™ (EC) software version 1.1 was used to evaluate the performance quality of the arrays including the labelling, hybridization, scanning and background signals. Further details regarding RNA isolation, processing and quality controls, including validation of array-based estimates of gene expression are reported in Trabzuni *et al.* (2011). Expression data on hESCs, neural precursors, terminally differentiated neurons and post-mortem adult brain samples have been submitted to Gene Expression Omnibus (accession number GSE34865).

### Analysis of exon array data

Exon Array data generated from hESCs (*n* = 5), neural precursors (*n* = 4), terminally differentiated neurons (*n* = 3), post-mortem adult brain samples originating from neuropathologically confirmed controls (*n* = 57) and foetal brain tissue (*n* = 94) were pre-processed using RMA quantile normalization with GC background correction in Partek’s Genomics Suite v6.6 (Partek Incorporated, St. Louis, MO, USA). Detection above background (DABG), *p* values of exon probe sets were calculated using Affymetrix Power Tools v1.14.3 (APT, http://www.affymetrix.com/partners_programs/programs/developer/tools/powertools.affx). Only probe sets with a median DABG *p* value of < 0.001 for any single cell/tissue type, a minimum of three probes within the set, unique hybridization and designed against genes annotated within Entrez Gene (http://www.ncbi.nlm.nih.gov/entrez/query.fcgi?db=gene) as documented in Netaffyx annotation file (HuEx-1_0-st-v2 Probe set Annotations, CSV Format, Release 31) were included in the analysis. Gene level summary signals were generated by calculating the Winsorized mean value (below 10% and above 90%) of all probe set signals annotated to a single gene (transcript cluster). As most exons are represented by only one probe set, we used the probe set signal intensity as synonym of exon expression level, unless explicitly mentioned. We defined an ‘expressed’ gene as any gene containing ≥ one exon with a median DABG *p* value < 0.001 in any given sample set. State-specific expression and splicing was investigated using Partek Genomic Suite’s mixed model anova and alternative splicing anova (Partek Genomics Suite v6.6). To reduce the likelihood of false positives, only probe sets that were called as present in both cell/tissue types being compared were included within the analysis. In all types of analysis, the date of array hybridization was included as a co-factor to eliminate batch effects. We used the FDR step-down method to correct *p* values for multiple comparisons. Conservative statistical thresholds were used to identify differentially expressed genes (FDR < 0.01 and minimum fold difference > 1.7 between sample sets) and differentially spliced genes (FDR < 1 × 10^−5^). Uncorrected *p* values are quoted within the text. Unsupervized hierarchical clustering, PCA and Gene Ontology analysis were performed using Partek Genomics Suite v6.6 (Partek Incorporated). GSEA was performed using GSEA v2.0.6 (Broad Institute, Cambridge, MA, USA) (Mootha *et al.* 2003; Subramanian *et al.* 2005). Enrichment score *p* values were estimated using an empirical phenotype-based permutation test procedure.

## Results

### Generation of hESC neural derivatives; NPCs and physiologically functional regional neurons

**Fig. 1:**
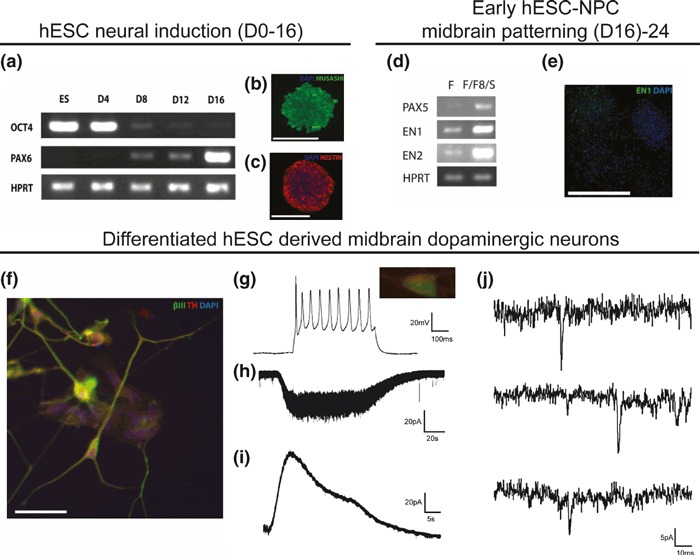
Specification of functional midbrain dopaminergic neurons from human embryonic stem cells (hESCs). (a–c) Neural induction from hESCs in chemically defined medium using a suspension culture system leads to progressive loss of pluripotency marker OCT4 and concomitant acquisition of neuroectodermal markers PAX6, MUSASHI and NESTIN by day 16. (d) The application of FGF8 and a sonic hedgehog agonist (puromorphamine) from day 16 to 24 increases the expression of PAX5, EN1 (Engrailed 1) and EN2, markers associated with midbrain dopaminergic neurons. (e–f) Terminal differentiation to a midbrain dopaminergic neuronal identity was demonstrated by EN1 expression and β-III-tubulin/Tyrosine hydroxylase (TH) co-immunolabelling. (g) Electrophysiological assessment of TH expressing neurons showed them to fire action potentials when injected with depolarizing currents. (h) In addition, they possessed receptors for ionotropic glutamate receptors, as evidenced by whole-cell current recordings in response to the application of α-amino-3-hydroxyl-5-methyl-4-isoxazole-propionate (AMPA). (i) Receptors for the inhibitory neurotransmitter, γ-aminobutyric acid (GABA) were also present in these cells. (j) Finally, synaptic connectivity was evident by the presence of miniature excitatory synaptic post-synaptic currents indicated by ‘*’. Scale bars: B&C 100 μm, E 250 μm, F 50 μm and G 10 μm.

Using well established differentiation protocols, we neurally converted OCT4 expressing pluripotent hESCS to first musashi, nestin and PAX6 expressing NPCs; and then, by application of FGF8 and a sonic hedgehog agonist (puromorphamine), to PAX5, EN1 (Engrailed 1) and EN2 expressing precursors (Fig. 1d and e). Importantly, these midbrain precursors plated at day 24 for a further 35 days (total days *in vitro* 59) generated highly enriched neurons that expressed ß-III-tubulin (84.1 ± 1.6%; Supporting Information Figure S1A), synapsin (89.4 ± 1.3%; Supporting Information Figure S1B) with evidence of a midbrain dopaminergic neuronal identity as demonstrated by EN1 expression (31.8.0 ± 2.0%; Fig. 1e and Supporting Information Figure S1C) and β-III-tubulin/Tyrosine hydroxylase (TH) co-immunolabelling (41.7.0 ± 3.1%; Fig. 1f and Supporting Information Figure S1D). Electrophysiological assessment of TH expressing neurons demonstrated firing of action potentials when injected with depolarizing currents (Fig. 1g). In addition, they possessed receptors for ionotropic glutamate receptors, as evidenced by whole-cell current recordings in response to the application of α-amino-3-hydroxyl-5-methyl-4-isoxazole-propionate (AMPA; Fig. 1h). Receptors for the inhibitory neurotransmitter, γ-aminobutyric acid (GABA), were also present in these cells (Fig. 1i). Finally, synaptic connectivity was evident by the presence of miniature excitatory synaptic post-synaptic currents. Together, these data are consistent with the generation of a subpopulation (approximately 30%) of functional human midbrain dopaminergic neurons from hESCs within a population of highly enriched neuronal cultures ( > 80%).

### Genome-wide gene expression analyses at developmentally distinct phases of lineage restriction

**Fig. 2:**
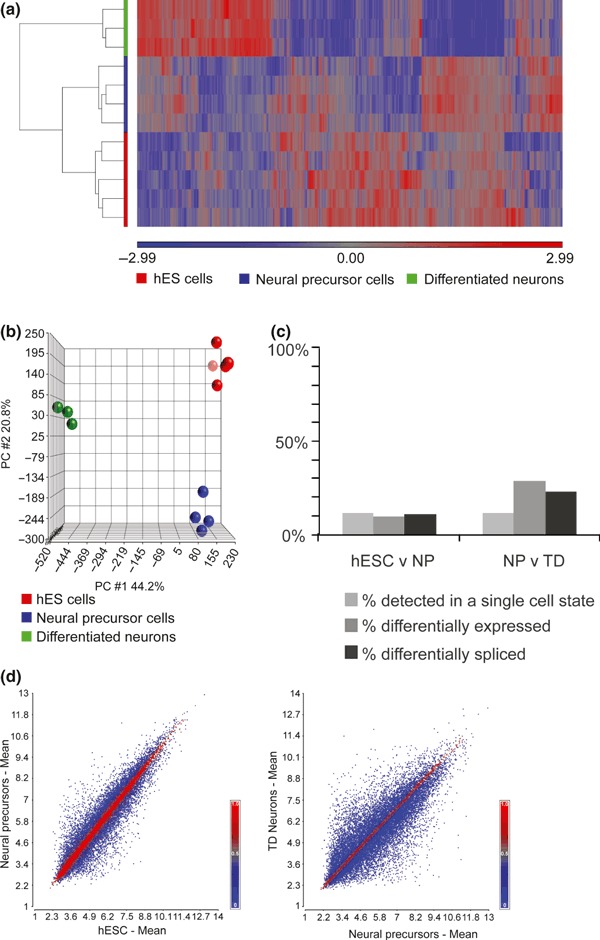
Gene expression profiling during neural differentiation in culture. (a) Unsupervized hierarchical clustering of hESC, NPCs and differentiated neuron exon-level expression profiles demonstrates separation of each cell type with human embryonic stem cells (hESCs) and NPCs profiles being most closely related. (b) Principal component analysis (PCA) confirms the separation of each cell type, hESC, NPCs and differentiated neurons. (c) Bar chart to show the % of all genes detected in two sequential stages of neural differentiation, either hESC and NPCs (hESC v NP) or NPCs and differentiated neurons (NP v TD), that are (i) only called present in one cell type, (ii) differentially expressed between cell types and (iii) differentially spliced between cell types. (d) Gene expression scatter plots comparing the hESC and NPC expression profiles, and the NPC and differentiated neuron expression profiles demonstrate that global gene expression in hESCs and NPC is more similar than NPCs and differentiated neurons.

To examine the relationship between hESCs, NPCs and terminally differentiated physiologically active neurons, whole-genome gene expression and mRNA splicing analysis using Affymetrix Exon arrays was undertaken. Unsupervized hierarchical clustering analysis and PCA revealed expected segregation of replicates with hESCs, NPCs and differentiated neurons forming distinct groups, with the hESC and NPC populations more closely related than differentiated neurons (Fig. 2a and b). To further characterize gene expression differences, the expression profiles (i) hESCs and NPCs and (ii) NPCs and differentiated neurons were next compared. This method detected genes expressed only in a single culture type, genes differentially expressed between cultures (as defined as a fold change of ± 1.7 and passing an FDR of 0.01) and genes, which were differentially spliced between cultures (passing an FDR of 1 × 10^−5^). This analysis confirmed the findings of hierarchical clustering by demonstrating that there were fewer gene expression differences between hESCs and NPCs, than between NPCs and differentiated neurons and also revealed that the majority of gene expression differences between NPCs and differentiated neurons were as a result of differential gene expression rather than expression of unique genes (Fig. 2c and d). Furthermore, this analysis demonstrated that differential splicing is common during neural differentiation and is likely to be no less important than–the more often studied–differential gene expression, particularly when comparing NPCs to differentiated neurons.

Using Gene Ontology analysis, we next focused on genes that passed an FDR of 0.01 and had at least 1.7-fold higher expression in (i) NPCs compared with hESCs, and (ii) terminally differentiated neurons as compared with NPCs. Consistent with previous reports, we found that genes involved in nervous system development (enrichment *p* value = 9.28 × 10^−10^) and synapse assembly (enrichment *p* value = 2.20 × 10^−9^) were significantly over-represented in NPCs compared to hESCs (Wu *et al.* 2010). Further enrichment of nervous system related genes was also apparent in terminally differentiated neurons compared to NPCs. Not only were genes involved in nervous system development (enrichment *p* value = 1.13 × 10^−12^) and synapse assembly (enrichment *p* value = 2.96 × 10^−9^) significantly over-represented in terminally differentiated neurons as compared to neural precursors, but so were genes involved more specifically in central nervous system development (enrichment *p* value = 3.80 × 10^−9^) and axon guidance (enrichment *p* value = 2.64 × 10^−10^). However, the most significant finding was the significant over-representation of genes related to cell adhesion (enrichment *p* value = 3.59 × 10^−25^).

**Fig. 3:**
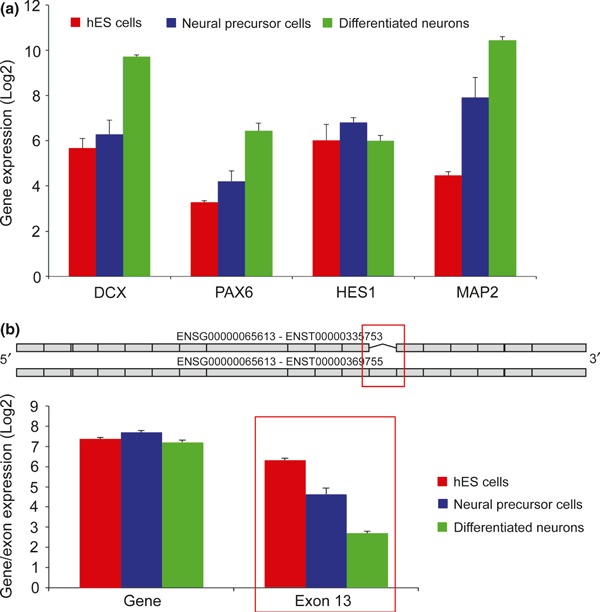
Dynamic gene expression and splicing during neural differentiation. (a) Bar chart showing *DCX*, *PAX6*, *HES1* and *MAP2* expression ± SEM in human embryonic stem cells (hESCs), NPCs and differentiated neurons. (b) Gene level and exon-specific expression data for *SLK* in hESCs, NPC cells and differentiated neurons demonstrates that while there is no significant difference in *SLK* gene level expression in hESCs, NPC cells and differentiated neurons, expression of exon 13 becomes significantly lower during neural differentiation (alternative splicing *p* value = 1.51 × 10^−22^).

Given data from previous studies demonstrating dynamic expression of *PAX6*, *DCX*, *MAP2* and *HES1* during neural differentiation, we investigated the expression of these genes in our own data set (Wu *et al.* 2010). Consistent with earlier reports, we found that expression of *PAX6*, *DCX* and *MAP2* increased significantly during neural differentiation (*PAX6 p* value = 7.83 × 10^−5^, *DCX p* value = 9.87 × 10^−5^, *MAP2 p* value = 2.86 × 10^−4^). *HES1* expression appeared to be maximal in NPCs (Fig. 3a).

Finally, we investigated alternative splicing during neuronal differentiation. Of the 13 625 multi-exon genes detected in both hESCs and neural precursors, 13.8% (1882) were identified as being alternatively spliced by cell type using a conservative FDR of 1 × 10^−5^. A similar analysis comparing NPCs and terminally differentiated neurons demonstrated that 29.9% (4324 of 14 440) of multi-exon genes detected in both samples had evidence of alternative splicing. An illustrative example is *SLK* where hESCs expressed the full-length isoform of *SLK* containing all exons, consistent with other studies (Yeo *et al.* 2007; Wu *et al.* 2010), and during the process of neural differentiation, the shorter isoform–lacking exon 13 – became dominant (Fig. 3b) (alternative splicing *p* value = 1.51 × 10^−22^, Supporting Information Figure S2). This appeared to be a gradual process such that NPCs expressed a mixture of both isoforms, whereas differentiated neurons expressed the short isoform of *SLK*. Thus, splicing is an important source of transcriptomic variation during the process of neural differentiation, no less important than the more frequently measured changes in gene expression levels.

### Comparison of hESC-derived neuronal cultures to human foetal and adult brain expression profiles

**Fig. 4:**
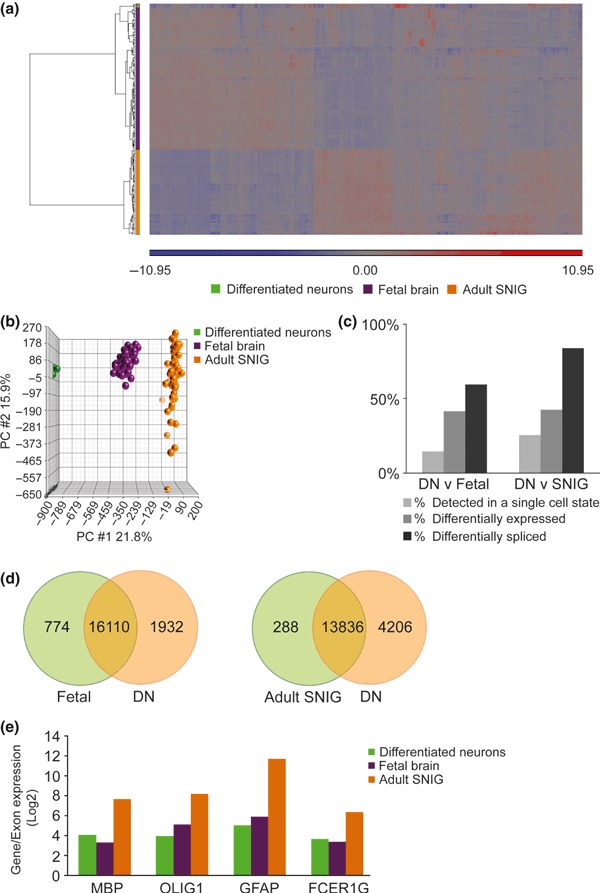
Gene expression profiling in differentiated neurons, foetal brain tissue and adult human substantia nigra. (a) Unsupervized hierarchical clustering of expression profiles from differentiated neurons, foetal brain tissue and adult substantia nigra demonstrates that while each cell/tissue type clusters separately, terminally differentiated neurons and foetal brain tissue expression profiles are most closely related. (b) Principal component analysis (PCA) confirms the separation of each cell/tissue type, differentiated neurons, foetal brain tissue and adult substantia nigra. (c) Bar chart to show the % of all genes detected in two sequential stages of neural differentiation, either differentiated neurons and foetal brain tissue (DN vs. Foetal) or differentiated neurons and adult substantia nigra (DN vs. SNIG), that are (i) only called present in one cell type, (ii) differentially expressed between cell types and (iii) differentially spliced between cell types. (d) Venn diagrams showing the overlap in genes called as expressed within differentiated neurons and foetal brain tissue (Foetal vs. DN), as compared with differentiated neurons and adult substantia nigra (Adult SNIG vs. DN). (e) Bar chart showing *MBP*, *OLIGO1*, *GFAP* and *FCER1G* expression ± SEM in differentiated neurons, foetal brain tissue and adult substantia nigra.

To relate the gene expression phenotype in hESC-derived neurons to the *in vivo* situation, we compared these data to archival data generated on the same platform from human foetal brain tissue (Johnson *et al.* 2009) and an additional data set generated within our laboratory from human adult substantia nigra (Trabzuni *et al.* 2011). The former data set consisted of samples originating from foetal cerebellum (*n* = 5), thalamus (*n* = 7), hippocampus (*n* = 8), striatum (*n* = 8) and neocortex (*n* = 66). Unsupervized hierarchical clustering and PCA revealed that the different populations segregated into the three ‘developmental’ groups, namely adult brain tissue, foetal brain tissue and hESC-derived neurons (Fig. 4a and b). Interestingly, when compared to both foetal brain samples (all regions) and adult brain, the differentiated neuronal (hESC-derived) cells appeared to be most closely related to foetal tissue samples, whatever their regional identity. As this would suggest that maturational state as opposed to regional identity was most significant, from this point on we refer to all foetal brain samples collectively as ‘foetal brain tissue’.

Closer inspection of the results would suggest that this finding was driven primarily by the greater overlap in expression of genes called as expressed between differentiated neuronal cultures and foetal brain tissue, as compared to neuronal cultures and adult brain (Fig. 4c and d). In both types of analysis, the majority of genes detected in a single state were expressed in differentiated neurons. Focusing on the gene sets uniquely expressed in differentiated neurons and using Gene Ontology analysis, we found an enrichment of genes involved in response to stimulus (enrichment *p* value = 2.39 × 10^−7^) and growth (enrichment *p* value = 0.013) in differentiated neurons versus foetal brain tissue, and in cell growth (enrichment *p* value = 1.23 x 10^5^), cell proliferation (enrichment *p* value = 0.003) and response to stimulus (enrichment *p* value = 0.043) in the differentiated neurons versus adult substantia nigra. Conversely, using Gene Ontology analysis to investigate genes *not* detected in neuronal cultures, but present in foetal and adult brain tissue, we found an enrichment of genes involved in cell–cell signalling (enrichment *p* value = 2.66 × 10^−9^), immune response (enrichment *p* value = 3.37 × 10^−9^ and multicellular organismal development (enrichment *p* value = 4.30 × 10^−9^). Further insights were obtained by looking specifically at genes associated with myelination (*MBP*), oligodendrocytes (*OLIG1*), astrocytes (*GFAP*) and microglia (*FCER1G*) (for the full list of genes tested see Supporting Information Table S2) (17). All four genes were expressed at significantly higher levels in adult substantia nigra (*MBP*, *p* value = 4.86 × 10^−14^, fold change = 12.08; *OLIGO1*, *p* value = 1.82 × 10^−23^, fold change = 18.58; *GFAP*, *p* value = 2.93 × 10^−21^, fold change = 100.34; *FCER1G*, *p* value = 4.50 × 10^−9^, fold change = 6.51) with very similar levels of expression in neuronal cultures and foetal brain tissue (Fig. 4e).

**Fig. 5:**
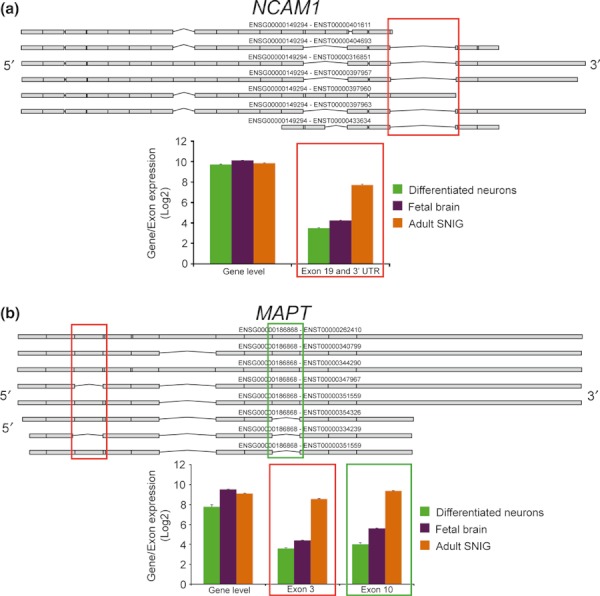
Differential splicing of *NCAM1* and *MAPT* in differentiated neurons, foetal brain tissue and adult substantia nigra. (a) Gene level and exon-specific expression data for *NCAM1* demonstrate that while there is no significant difference in *NCAM1* gene level expression in differentiated neurons, foetal brain tissue or adult substantia nigra, expression of the 3′ UTR specific to NCAM-120 has a significantly higher expression in adult substantia nigra as compared to foetal brain tissue or differentiated neurons (alternative splicing *p* value < 1.0 × 10^−45^). (b) Gene level and exon-specific expression data for *MAPT* demonstrate that while *MAPT* at the gene level is expressed at high levels in differentiated neurons, foetal brain tissue and adult substantia nigra, expression of exons 2 and 10 is significantly higher expression in adult substantia nigra as compared to foetal brain tissue or differentiated neurons (alternative splicing *p* value < 1.0 × 10^−45^).

We also identified genes that were (i) differentially expressed between conditions, as defined as a fold change of ± 1.7 between states and passing an FDR of 0.01, and (ii) genes which while they might have been expressed at similar levels between states were differentially spliced as defined as passing an FDR of 1 × 10^−5^ using the alternative splicing anova. Together these findings show that differential splicing is an important source of variation between hESC-derived neuronal cultures and *in vivo* tissues both foetal and adult. Indeed, the finding that differential splicing was the most frequent observation between hESC-derived neurons and adult tissue emphasizes the significance of maturation-dependent differential splicing (Fig. 4c). Illustrative analysis of NCAM1 and MAPT, two well-studied genes that are known to be spliced in a developmentally regulated manner, differential splicing (as opposed to differential gene expression) made it possible to distinguish between adult, as compared to foetal or hESC-derived cell samples (Fig. 5a and b). For example, *NCAM1* is spliced to generate three main isoforms, the protein products of which are commonly known as NCAM-180, NCAM-140 and NCAM-120. Whereas NCAM-180 and NCAM-140 have the highest expression during foetal and early post-natal development, NCAM 120 is found at low levels in foetal brain, but increases in expression and is expressed at stable levels in adult (Cox *et al.* 2009). In keeping with these findings, we found that expression of the 3′UTR (as measured by three probe sets on the Affymetrix Exon Array) unique to NCAM-120 were expressed at similar levels in differentiated neurons and foetal brain tissue, but had higher expression in adult brain (alternative splicing *p* value < 1.0 × 10^−45^) (Fig. 5a, Supporting Information Figure S3). Similarly, *MAPT* is alternatively spliced at exons 3 and 10, and analysis of the differentiated neuronal cells and foetal tissue show that both of these exons are expressed at significantly lower levels compared to the adult brain (alternative splicing *p* value < 1.0 × 10^−45^) (Fig. 5b, Supporting Information Figure S4). This is in accordance with previous developmental data and data from a previously published study from us (Andreadis 2005; Iovino *et al.* 2010).

### Gene set enrichment analysis to specifically investigate synapse formation

**Fig. 6:**
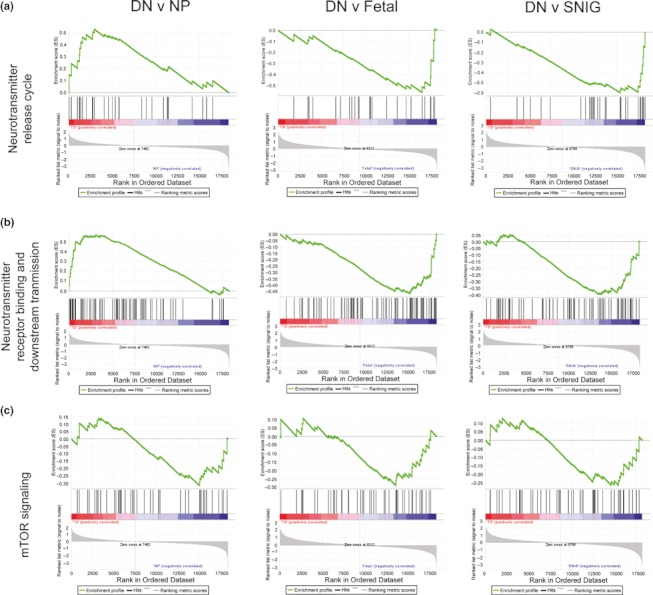
Gene set enrichment analysis (GSEA) for three pathways relevant to neural differentiation. (a) GSEA for the Reactome neurotransmitter release cycle demonstrates significant enrichment of this pathway in differentiated neurons as compared to NPC cells (DN vs. NP), in addition to enrichment of this pathway in foetal brain tissue and adult substantia nigra relative to differentiated neurons (DN vs. Foetal, DN vs. SNIG). (b) GSEA for the Reactome neurotransmitter receptor binding and downstream transmission pathway demonstrates significant enrichment of this pathway in differentiated neurons as compared to NPC cells (DN vs. NP), in addition to enrichment of this pathway in foetal brain tissue and adult substantia nigra relative to differentiated neurons (DN vs. Foetal, DN vs. SNIG). (c) GSEA for the KEGG mTOR signalling pathway provides no evidence for significant enrichment of this pathway in neural precursors, foetal brain tissue or adult substantia nigra on comparison with differentiated neurons (DN vs. NP, DN vs. Foetal, DN vs. SNIG).

Investigating the differential expression of single genes will underestimate differences in the expression of entire gene networks that are subtle at the level of any single gene, but are nonetheless significant when all the genes in the pathway are considered. To address this issue, we used the Broad Institute’s Molecular Signatures Database and GSEA software (v2.0.6) to identify gene sets related to pre- and post-synaptic signalling, namely the Reactome ‘neurotransmitter release cycle’ and ‘neurotransmitter receptor binding and downstream transmission in the post-synaptic cell’ (Fig. 6). GSEA revealed that both pathways are up-regulated in differentiated hESC-derived neurons as compared to NPCs (Reactome ‘neurotransmitter release cycle’, nominal *p* value = 0.012, FDR *q* value = 0.007; Reactome ‘neurotransmitter receptor binding and downstream transmission in the post-synaptic cell’, nominal *p* value < 1.0 × 10^−3^, FDR *q* value = 1.0 × 10^−3^). However, investigating the expression of these pathways in foetal brain tissue (Reactome ‘neurotransmitter release cycle’, nominal *p* value = 0.003, FDR *q* value = 0.005; Reactome ‘neurotransmitter receptor binding and downstream transmission in the post-synaptic cell’, nominal *p* value = 1.0 × 10^−3^, FDR *q* value = 0.009) and adult substantia nigra (Reactome ‘neurotransmitter release cycle’, nominal *p* value = 0.005, FDR *q* value = 0.003; Reactome ‘neurotransmitter receptor binding and downstream transmission in the post-synaptic cell’, nominal *p* value = 0. 0.029, FDR *q* value = 0.059) demonstrated that both pathways are more highly expressed in these tissue types than neuronal cultures. This would suggest that while hESC-derived neurons are capable of releasing and responding to neurotransmitters, neither the pre-synaptic nor post-synaptic machinery is expressed at foetal or adult levels. Conversely, a similar analysis investigating the KEGG mTOR signalling pathway demonstrated no enrichment of this pathway between hESC-derived differentiated neurons and NPCs (nominal *p* value = 0.367, FDR *q* value = 0.381), differentiated neurons and foetal brain tissue (nominal *p* value = 0.316, FDR *q* value = 0.334), and finally differentiated neurons and adult brain tissue (nominal *p* value = 0.703, FDR *q* value = 0.718) (Fig. 6). This would suggest that this pathway matures early and is expressed at similar levels in all three maturational states.

## Discussion

Human pluripotent cells represent a potentially limitless source of neurons for further study, both as an experimental resource and potentially as a cell-based repair strategy in neurodegenerative disease. A major limitation to using human stem cell derivatives as either a therapeutic treatment or as model systems for examining neurodegenerative diseases is the relative lack of information regarding the functional and molecular maturational state of differentiated neuronal populations in relation to the ageing human brain (Lees *et al.* 2009). One way of approaching this is to examine alterations in expression of genes over the course of the differentiation process and in reference to human foetal and adult brain tissue. Advances in genome-wide array technology allow an unbiased analysis of the entire genome, with both gene and exon-specific expression quantified. In this study, we have analysed whole-genome gene expression using an exon array platform, yielding a detailed map of gene expression and splicing in three temporally distinct cellular stages in the process of neural conversion from pluripotent stem cells. This allowed us to compare these gene expression phenotypes to those observed in the human foetal and adult brain and thus characterize their utility in modelling neurodegenerative disease *in vitro*.

The analysis presented in this article represents the first report of genome-wide mRNA expression and splicing during the process of directed differentiation from hESCs to a terminally differentiated neuronal population. It provides a platform for the analysis of targeted differentiation of pluripotent human cells into defined neuronal populations, related to their *in vivo* counterparts. Analysis of our mRNA expression data from the hESC, NPC and differentiated neuronal populations confirms that the phenotypic changes in the properties and function of cell populations cultured *ex vivo* can be followed via the medium of gene expression, a finding that is consisistent with previous genome-wide expression studies of this nature (Yeo *et al.* 2007; Wu *et al.* 2010; Fathi *et al.* 2011). As the differentiation process is followed, there is increasing disparity between the expression profiles of these cells, with greater difference observed between NPCs and differentiated neuronal cells than between hESCs and NPCs – demonstrating an overlap within precursor or cycling cell populations and increased specialization upon lineage restriction. This is confirmed by unsupervized hierarchical clustering and PCA; the three cell populations can be clearly separated, with each set displaying close grouping suggesting that the differentiation process results in reproducible populations.

These data highlight the utility of genome-wide expression analysis as a quality control measure for following differentiation and assessing the robustness of a differentiation protocol. A detailed analysis of the type of expression differences between the cell cultures revealed that although expression of unique genes and differential gene expression is important, differential splicing is also a major cause of transcriptomic variation particularly when comparing NPCs to neuronal cultures. In the case of both differential gene expression and splicing, we were able to replicate and expand on a previous study using RNA sequencing of hESCs (Wu *et al.* 2010). Similarly, we showed that hESCs express the full-length isoform of SLK and that during the process of neural differentiation, the shorter isoform lacking a single exon becomes dominant. As SLK splicing has been previously demonstrated using both RNA sequencing (Wu *et al.* 2010) and qPCR (Yeo *et al.* 2007), this example also provides strong validation for the splicing changes predicted using the Affymetrix Exon array.

An insight into the nature of increasing complexity in neural tissue as it develops is yielded by our analysis of the differential expression and splicing of genes between sample sets. A key finding of this study is that, in addition to differential gene expression, differential splicing is a common finding. This highlights the importance of altered splicing in establishing the complexity and diversity of a fully formed neuronal system, as well as the importance of assessing splicing as a measure of expression phenotype in hESC-derived neuronal populations used as model systems. Although it might be argued that differential splicing between neuronal cultures and *in vivo* samples is being overestimated possibly because of the higher cellular diversity within foetal brain tissue and adult brain, it should be noted that only exons called as present within all sample types were analysed for evidence of alternative splicing; thus reinforcing the validity of our findings.

Focusing on the terminally differentiated hESC-derived neurons, foetal and adult brain data sets, the groups of genes that are uniquely expressed by the hESC-derived neuronal cultures are informative as to the nature of the differences between these samples. The clearest difference between the hESC-derived neurons and the foetal brain samples is in genes linked to response to stimulus and growth, suggesting that growth-related signalling is still highly active within the cultures. Between the hESC-derived neurons and adult brain data set, growth and cell proliferation standout as major gene groups that are differentially expressed between the two sample sets. However, it is also clear that at least some of the differences between neuronal cultures and adult brain (in particular) arise from the expression of cellular systems and types that are not present in culture systems. Among the genes *not* expressed in cultures, but called present *in vivo,* there was a clear enrichment of genes associated with cell–cell signalling, immune response and multicellular organismal development.

Similarly, focusing on the expression of genes specifically associated with myelination, oligodendrocytes, astrocytes and microglia demonstrated the higher expression of these cell types in adult brain as compared with both foetal tissue and neuronal cultures. The expression data presented in this article comes from mixed cell populations, both with regard to the differentiated cellular samples and the post- mortem samples. In particular, and as demonstrated in previous similar studies (Li *et al.* 2005; Joannides *et al.* 2007; Patani *et al.* 2011), the hESC-derived neuronal population is likely to contain a higher neuron ( ≥ 85%) to glia ( ≤ 5–10%) ratio when compared with the post-mortem samples (where glia are likely to outnumber neurons). Some of the genome-wide gene expression differences identified between hESC-derived neurons and their foetal and adult counterparts may well reflect their different lifespan and developmental environment/signalling milieu. It may indeed be of interest in future studies to employ *ex vivo* cultured adult neuronal primary cells (e.g. from brain biopsy material) to compare expression profile differences with their *in vitro* hESC-derived counterparts. A direct comparison between primary neuronal populations and ESC derived neuronal cells could also be achieved under more controlled conditions using murine derived cells. In terms of more closely modelling the *in vivo* situation, establishing carefully controlled hESC-derived co-cultures (neurons and glia) may enable more representative comparisons to be made to the post-mortem adult brain on a cell population level. This will be of particular importance with regard to studying the non-cell autonomous aspects of neuronal degeneration, as evidenced by experimental data from familial forms of amyotrophic lateral sclerosis (Ilieva *et al.* 2009).

When expression data from hESC-derived neuronal cultures (containing approximately 30% midbrain dopaminergic neurons) is compared with profiles from human foetal brain tissue and adult brain (substantia nigra), there is clear clustering of the expression data set from these samples with foetal brain tissue, highlighting the expression phenotype similarities between these data sets. The finding of comparative maturational equivalence between hESC-derived neuronal populations and foetal (not adult) brain tissue has important implications for stem cell-based approaches to modelling neurological disease. Notwithstanding that recent human iPSC studies have reported phenotypes for both inherited and sporadic adult brain disorders, our findings raise important questions and offer insights around how adult-onset neurogenetic diseases can be optimally modelled *in vitro* using patient-derived, disease-specific induced pluripotent stem cells (iPSCs) (Wichterle and Przedborski 2010; Brennand *et al.* 2011; Nguyen *et al.* 2011; Bilican *et al.* 2012). In contrast to neuro-developmental disorders such as spinal muscular atrophy (Ebert *et al.* 2009), familial dysautonomia (Lee *et al.* 2009) and Rett’s syndrome (Marchetto *et al.* 2010), our study suggests strongly that modelling the adult context of disease requires a more complex culture model that seeks to reflect both glial enrichment and simulation of age-related pathological changes.

The purpose of our study was to identify differences in gene expression between currently used human pluripotent stem cell-based differentiation protocols and their somatic counterparts at later developmental stages to provide insight into the validity of current stem cell-based approaches to model neurological disease. This study highlights important differences between current *in vitro* strategies attempting to model neurodegeneration and the *in vivo* adult context. Our findings provide a platform for future strategies aiming to resolve the molecular pathogenesis of ageing and neurodegeneration, for example in conditions such as Parkinson’s and Alzheimer’s diseases.

In conclusion, our analyses suggest a potential hierarchical paradigm where developmental stage plays a greater role in regulating gene expression than regional identity of neuronal populations. The finding that the maturational state of hESC-derived terminally differentiated neurons is closer to that of foetal (not adult) neurons highlights the current limitations of *in vitro* modelling of adult neurodegenerative disease. In turn, this raises various experimental opportunities around ‘accelerating’ the developmental stage of neuronal cultures to more closely resemble their adult counterparts, and thus serve as more representative *in vitro* model systems to study human age-related neurological disease.

## Abbreviations

CDM: chemically defined medium
DABG: detection above background
EN1: engrailed 1
GSEA: gene set enrichment analysis
hESCs: human embryonic stem cells
iPSCs: induced pluripotent stem cells
PCA: principal component analysis
TH: tyrosine hydroxylase

## Supporting information

**Figure S1.** Quantitative immunocytochemistry for midbrain dopaminergic neuron specification from hESCs.

**Figure S2.** Plotted are expression levels (*y*-axis) for each probeset (*x*-axis) for *SLK*, which shows astatistically significant interaction between probeset expression (‘exon usage’) and cell type.

**Figure S3.** Plotted are expression levels (*y*-axis) for each probeset (*x*-axis) for *NCAM1*, which shows astatistically significant interaction between probeset expression(‘exon usage’) and cell/tissue type.

**Figure S4.** Plotted are expression levels (*y*-axis) for each probeset (*x*-axis) for *MAPT*, which shows astatistically significant interaction between probeset expression (‘exon usage’) and cell/tissue type.

**Table S1.** Sample details.

**Table S2.** Table to show the genes and their associated cell types and processes that have been tested for significant changes in expression between neuronal cultures and fetal brain tissue or adult substantia nigra.

As a service to our authors and readers, this journal provides supporting information supplied by the authors. Such materials are peer-reviewed and may be re-organized for online delivery, but are not copy-edited or typeset. Technical support issues arising from supporting information (other than missing files) should be addressed to the authors.
