# Supplementary material for: Investigating the utility of human embryonic stem cell-derived neurons to model ageing and neurodegenerative disease using whole-genome gene expression and splicing analysis
Source: J Neurochem. 2012 Aug;122(4):738–51. doi: 10.1111/j.1471-4159.2012.07825.x (PMC3504076; doi:10.1111/j.1471-4159.2012.07825.x)

Investigating the utility of human embryonic stem cell-derived neurons to model ageing and neurodegenerative disease using whole genome gene expression and splicing analysis

Rickie Patani, Patrick A. Lewis, Daniah Trabzuni, Clare A. Puddifoot, David J. A. Wyllie, Robert Walker, Colin Smith, Giles E. Hardingham, Michael Weale, John Hardy, Siddharthan Chandran and Mina Ryten

SUPPLEMENTARY DATA

Supplementary Table 2

Supplementary Figure 1

Supplementary Figure 2

Supplementary Figure 3

Supplementary Figure 4

Supplementary Table 2: Table to show the genes and their associated cell types and processes that have been tested for significant changes in expression between neuronal cultures and fetal brain tissue or adult substantia nigra.

Cell type/ Process	Gene Symbol	Transcript ID	p-value(Fetal brain tissue vs. Neuronal cultures)	Fold change(Fetal brain tissue vs. Neuronal cultures)	p-value(Adult SNIG vs. Neuronal cultures)	Fold change(Adult SNIG vs. Neuronal cultures)
Myelination	MAG	3830320	0.0870749	0.73	1.64E-36	29.78
Myelination	MBP	3814063	0.0605244	0.58	4.86E-14	12.08
Myelination	MOG	2900940	0.259801	0.83	1.00E-45	86.51
Myelination	PLP1	3985717	2.27E-07	0.14	1.64E-06	6.12
Myelination	C11orf9	3333169	2.12E-33	0.05	0.24867	0.81
Astrocytes	ALDOC	3750767	1.71E-14	0.08	1.54E-06	4.37
Astrocytes	GFAP	3759410	0.13316	1.82	2.93E-21	100.34
Astrocytes	S100B	3935486	1.35E-09	0.21	3.35E-21	15.93
Microglia	CFHR3	2373406	0.0695001	0.73	3.06E-09	2.97
Microglia	FCER1G	2363562	0.512654	0.82	4.50E-09	6.51
Microglia	TNIP2	2757944	0.035067	0.75	0.954313	1.01
Oligodendrocytes	CNP	3721548	8.60E-06	0.58	2.53E-22	4.15
Oligodendrocytes	OLIG1	3918429	0.000804406	2.22	1.82E-23	18.58
Oligodendrocytes	OLIG2	3918369	1.44E-06	2.80	5.90E-15	6.24
Oligodendrocytes	PDGFRA	2727226	0.405425	1.12	0.000500917	1.65
Oligodendrocytes	CSPG4	3633578	2.32E-20	0.34	0.000161149	0.68

Supplementary Figure 1: Quantitative immunocytochemistry for midbrain dopaminergic neuron specification from hESCs. Midbrain precursors plated at day 24 generated highly enriched neurons that expressed ß-III-tubulin (84.1±1.6%; A) and synapsin (89.4±1.3%; B). Terminal differentiation to a midbrain dopaminergic neuronal identity was demonstrated by EN1 expression (31.8.0±2.0%; C) and βIII-tubulin/TH co-immunolabeling (41.7.0±3.1%; D).

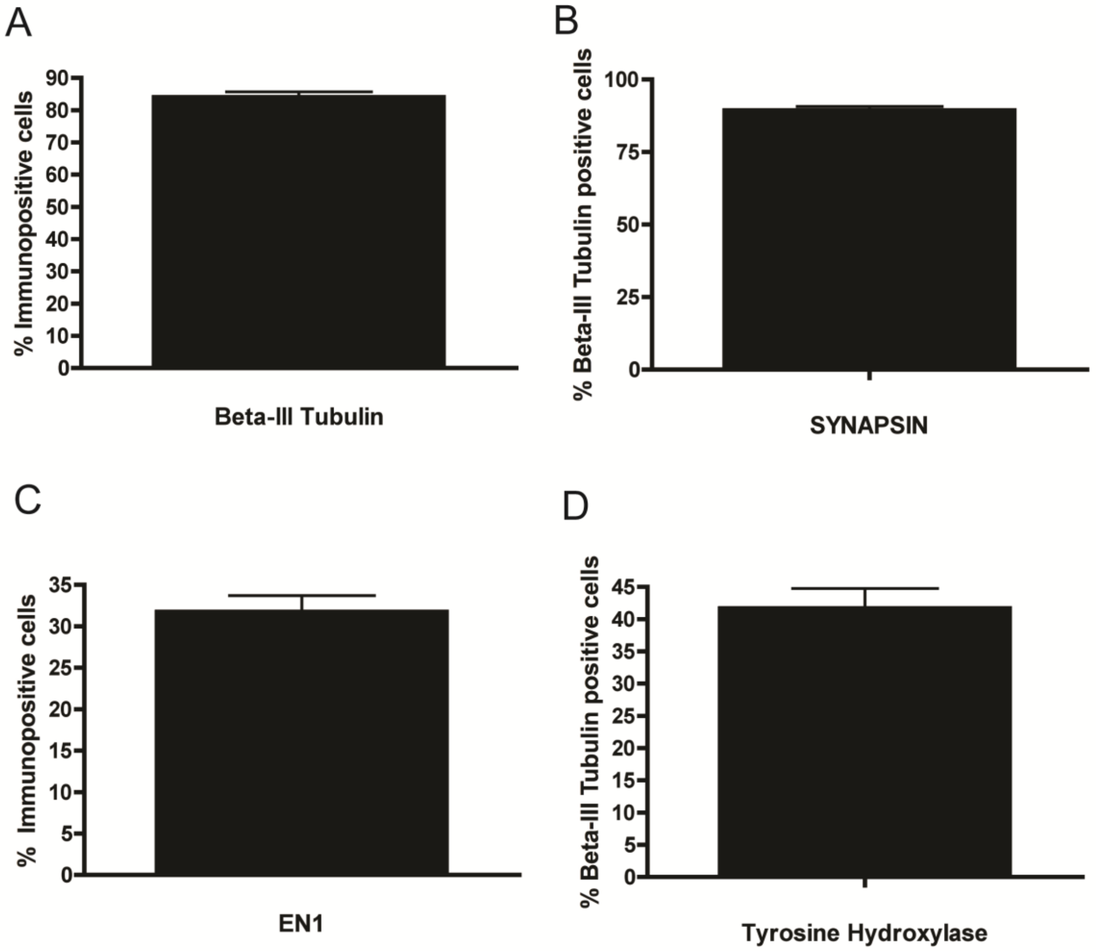

Supplementary Figure 2: Plotted are expression levels (y-axis) for each probeset (x-axis) for *SLK*, which shows a statistically significant interaction between probeset expression ("exon usage") and cell type. Non-parallel probeset expression levels (highlighted in the boxed region) indicate cell type-dependent differential splicing of the corresponding exon. Plots are adapted from Partek Genomics suite auto-generated output.

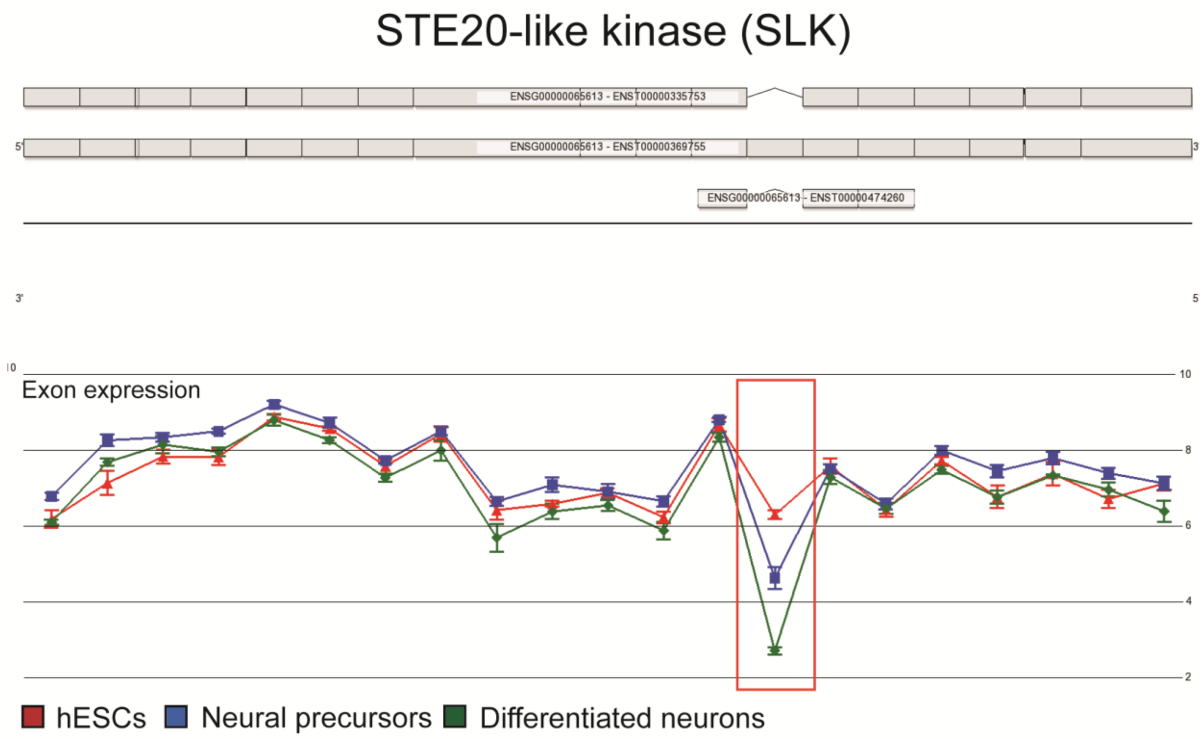

Supplementary Figure 3: Plotted are expression levels (y-axis) for each probeset (x-axis) for *NCAM1*, which shows a statistically significant interaction between probeset expression ("exon usage") and cell/tissue type. Non-parallel probeset expression levels (highlighted in the boxed region) indicate cell/tissue type-dependent differential splicing of the corresponding 3'UTR. Plots are adapted from Partek Genomics suite auto-generated output.

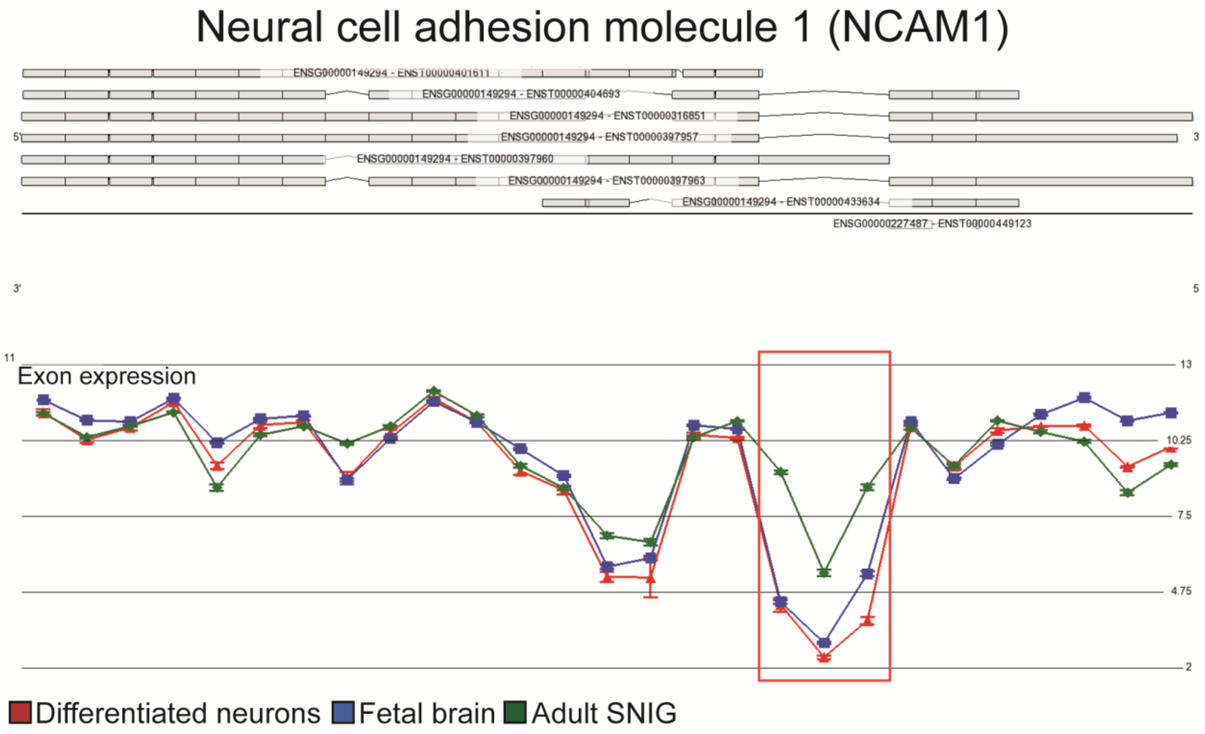

Supplementary Figure 4: Plotted are expression levels (y-axis) for each probeset (x-axis) for *MAPT*, which shows a statistically significant interaction between probeset expression ("exon usage") and cell/tissue type. Non-parallel probeset expression levels (highlighted in the boxed regions) indicate cell/tissue type-dependent differential splicing of the corresponding exons. Plots are adapted from Partek Genomics suite auto-generated output.

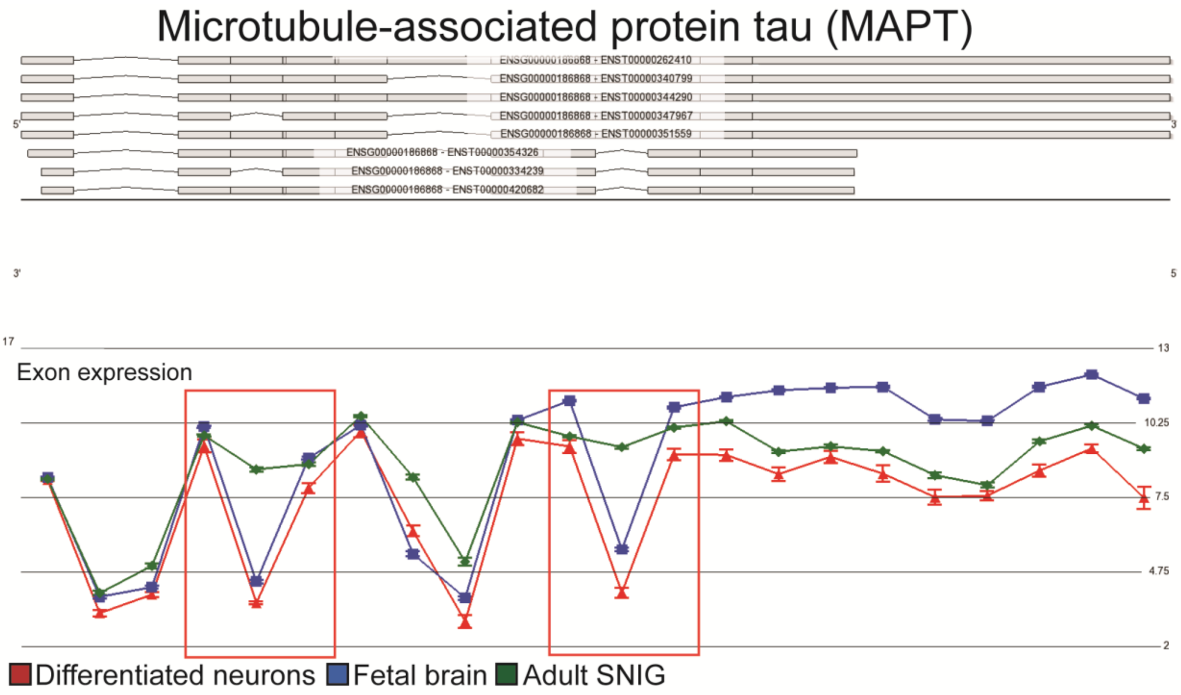

Supplement: Supplementary file 2 [file jnc0122-0738-SD2.pdf]
